# Primary endobronchial melanoma: a case report and clinical management indications

**DOI:** 10.1186/s12890-024-02904-2

**Published:** 2024-02-24

**Authors:** Emanuela Barisione, Andrea Boutros, Marco Mora, Francesco Spagnolo, Enrica Teresa Tanda, Carlo Genova, Elena Tagliabue

**Affiliations:** 1 Interventional Pulmonology Unit, IRCCS Ospedale Policlinico San Martino, Genova, Italy; 2 Department of Internal Medicine and Medical Specialties (DiMI), School of Medicine, University of Genoa, Genova, Italy; 3 Skin Cancer Unit, IRCCS Ospedale Policlinico San Martino, Oncologia Medica 2, Genova, Italy; 4 U.O. Anatomia Patologica Ospedaliera, IRCCS Ospedale Policlinico San Martino, Genova, Italy; 5 Department of Surgical Sciences and Integrated Diagnostics (DISC), Plastic Surgery Division, University of Genoa, Genova, Italy; 6 UOC Clinica di Oncologia Medica, IRCCS Ospedale Policlinico San Martino, Genova, Italy

**Keywords:** Melanoma, Endobronchial, Lung, Mucosal, Bronchoscopy, Endoscopy, Immunotherapy, Pembrolizumab, Nivolumab, BRAF, Case report

## Abstract

**Background:**

While cutaneous melanomas are well-documented, primary melanoma of the lung (PMML), particularly with endobronchial origin, remains rare and poorly characterized. This case report addresses gaps in understanding by presenting a comprehensive case of a 71-year-old male with primary endobronchial melanoma and conducting a systematic review of PMML cases.

**Case Presentation:**

The patient, a former smoker, presented with dyspnea, cough, and hemoptysis. Imaging revealed left lung atelectasis and a suspicious nodule. Bronchoscopy identified an endobronchial mass, subsequently treated with argon plasma coagulation and resection. Biopsy confirmed melanoma. Extensive examinations ruled out a primary skin lesion. Despite initial treatment, recurrence led to pneumonectomy. Histopathology confirmed melanoma. The patient received treatment with pembrolizumab and ipilimumab, but with poor clinical benefit.

**Conclusions:**

Primary endobronchial melanoma is a rare entity, comprising 0.01% of lung tumors. This case underscores diagnostic challenges and emphasizes histological criteria to distinguish primary from metastatic lesions. The pathogenesis remains unclear, with theories proposing foetal melanocyte migration or squamous metaplasia. Prognosis varies, necessitating radical surgical extirpation. A systematic review revealed diverse outcomes, supporting the need for further research. In conclusion, endobronchial melanoma involves an endoscopic and surgical management, but evolving therapies, such as immunotherapy, may reshape treatment paradigms. This case contributes to our understanding of PMML, guiding future research and clinical management. As therapeutic options evolve, continued research is crucial to refine our understanding and improve outcomes for this rare malignancy.

## Background

Melanoma, primarily associated with the skin, is a malignancy originating from melanocytes [1]. Although it typically affects the tegumentary system, melanoma can also develop in various body locations [1]. The respiratory system, frequently affected by aggressive malignancies like non-small cell lung cancer and mesothelioma, rarely encounters primary melanoma within its tract [2].

Primary extrapulmonary tumours often metastasize to the lung parenchyma but infrequently to the airways [2]. By 2004, literature spanning four decades had only described 204 cases of endobronchial metastases [3]. Malignant melanoma accounts for 4.5% of these metastases from extra-thoracic primaries [2]. The scarcity of primary melanoma of the lung (PMML), particularly with endobronchial origin, is highlighted by the limited case studies and series available, leaving many of its aspects under-characterized.

The incidence, pathological features, clinical behaviour, and optimal treatment strategies for these melanomas remain unclear [4]. Some reported cases, managed decades ago with outdated methods, fail to reflect current diagnostic and therapeutic advancements. Notably, there has been a lack of recent systematic reviews synthesizing the knowledge on this rare malignancy, despite the existence of sporadic case reports.

In an effort to fill these knowledge gaps, we present a case of a 71-year-old man with primary endobronchial melanoma and perform a comprehensive review of the literature in PubMed. Our goal is to consolidate current information on endobronchial primary melanoma, offering a detailed perspective on its epidemiology, clinical presentation, histopathology, diagnostic challenges, and treatment options. This work seeks to enhance understanding of PMML and to inform future research and clinical practice.

## Case presentation

**Fig. 1:**
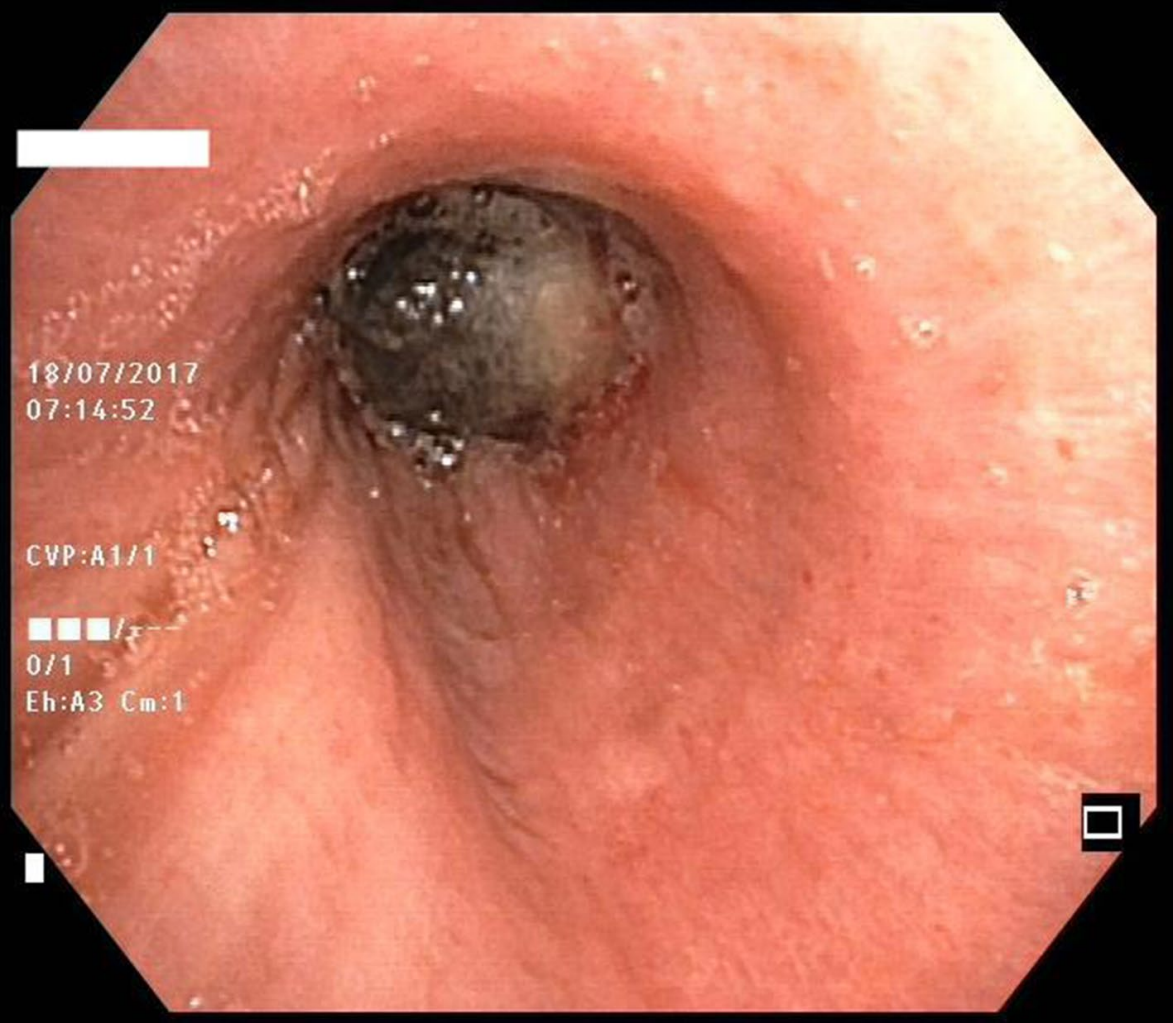
Initial flexible bronchoscopy identifying a complete stenosis of the left main bronchus, caused by a highly vascularized, dark, polypoid endobronchial mass

A 71-year-old Caucasian male, a former smoker, presented with worsening dyspnoea and cough accompanied by occasional haemoptysis over a two-month period. He had no significant medical history. Radiographic assessment revealed complete atelectasis of the left upper lobe and partial atelectasis of the left lower lobe. A comprehensive diagnostic workup, including total body computed tomography (CT) and total body positron emission tomography (PET), showed a suspicious left lung nodule. A subsequent flexible bronchoscopy revealed complete stenosis of the left main bronchus caused by a highly vascularized, dark, polypoid endobronchial mass (Fig. 1). Emergency argon plasma coagulation and mechanical resection with a rigid bronchoscope were performed to restore airway patency. The primary aim of this initial treatment was to improve the patient’s performance status sufficiently for potential systemic therapy.

**Fig. 2:**
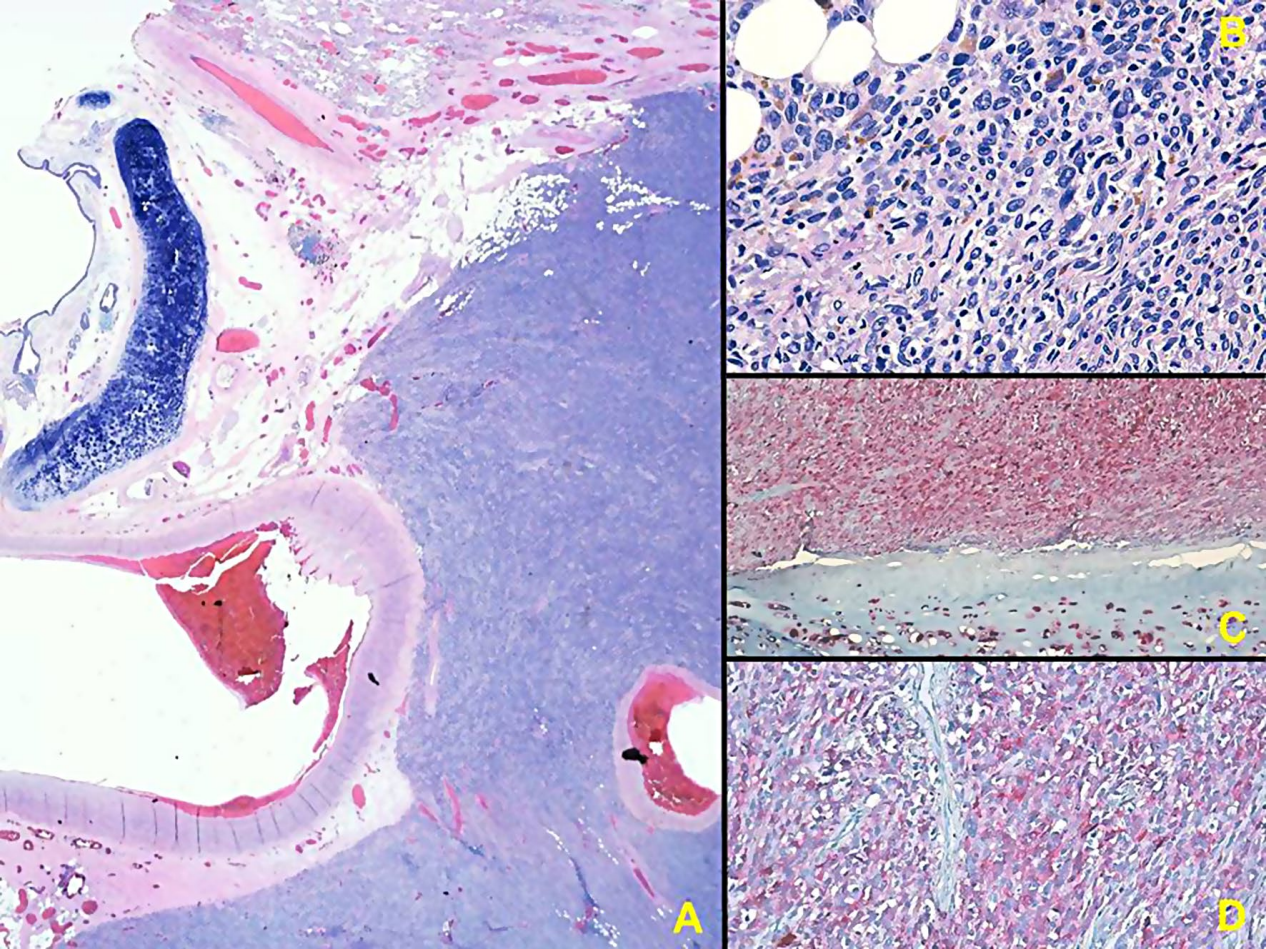
Neoplastic cells exhibited characteristic features, including medium-sized atypical epithelioid and spindle cells with eosinophilic cytoplasm, voluminous nuclei, and prominent nucleoli (**A**-**B**). Expression of pS100, Melan-A, and HMB-45 markers confirmed the melanocytic origin, with the absence of keratins AE1/AE3, confirming the diagnostic hypothesis of melanoma (**C**-**D**)

Histological examination of bronchial biopsies revealed necrosis and fragments of bronchial mucosa involved by a neoplastic proliferation of atypical epithelioid and spindle cells of medium size. These cells were characterized by eosinophilic cytoplasm and voluminous nuclei, often with prominent nucleoli. Immunohistochemistry confirmed the expression of pS100, Melan-A, and HMB-45 markers in the neoplastic cells, with no expression of keratins AE1/AE3. This led to the diagnosis melanoma (Fig. 2).

The patient denied any history of skin lesion removal in the past. Extensive examinations of the skin, eyes, anorectal region, genitourinary tract, and oesophagus failed to reveal occult primary lesions. From September 2017 to April 2018, the patient underwent pembrolizumab therapy.

A CT scan conducted three months later showed a re-occlusion of the left main bronchus, leading to another session of argon plasma coagulation and mechanical resection with a rigid bronchoscope. A biopsy of the main bronchus in presumably healthy tissue was also performed, which yielded negative results for neoplastic cells. Subsequently, the patient underwent a pneumonectomy.

**Fig. 3:**
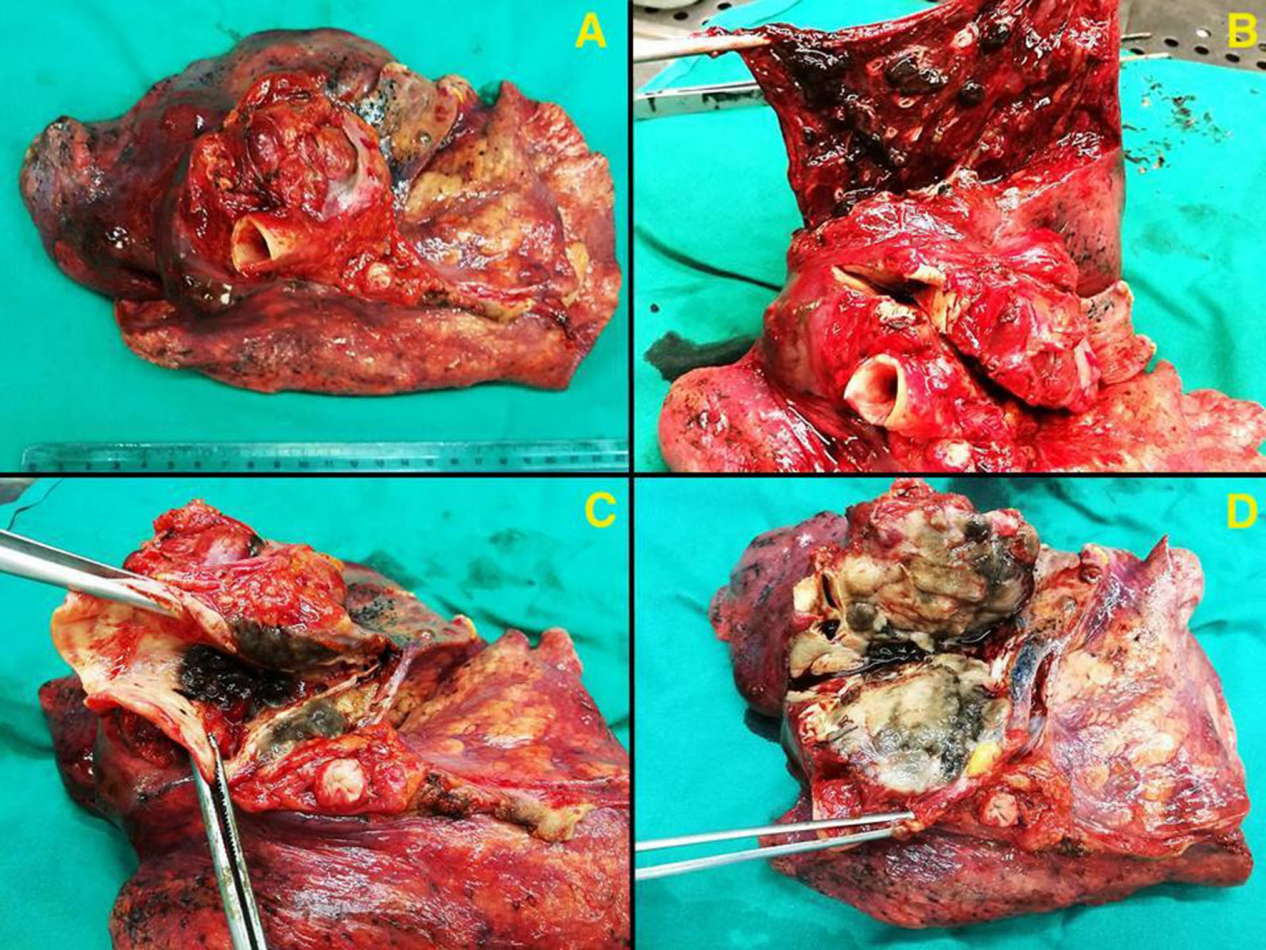
Gross pathology of left lung melanoma: Panel **A** presents an overview of the resected mass from the left lung’s hilum. The tumour, measuring 7 × 5 × 8 cm, appears as a greyish-black mass with heterogeneous consistency (**B**-**D**). Panel B clearly shows the melanoma’s pigmentation and heterogeneity. In Panel **C**, the forceps lift a section to reveal the tumour’s depth and its infiltration into the lung tissue. Panel **D** emphasizes the extensive involvement of the tumour, with clear encasement of the main and upper lobe bronchi, as well as extension into the lung parenchyma, regional lymph nodes, and adipose tissue of the hilum. These images highlight the extent of disease progression and the complex surgical challenge presented by the tumour’s location and involvement of multiple structures

A left pneumonectomy was performed, with the entire specimen weighing 480 g. The upper lobe measured 10 × 8 × 7 cm, and the lower one measured 18 × 10 × 8 cm. Gross examination revealed a neoplastic mass in the hilar region of the left lung, measuring 7 × 5 × 8 cm, with a greyish-black appearance at the cut surface. The tumour encased the main bronchus, the upper lobe bronchus, and extended into the lung parenchyma, lymph nodes, and adipose tissue of the hilum (Fig. 3).

Microscopic examination revealed that the mass was composed of solid sheets of atypical epithelioid and spindle cells of medium size, with areas of necrosis and haemorrhage. Intracellular brown pigment, indicative of melanin, was observed. Immunohistochemistry confirmed the expression of pS100, Melan-A, and HMB-45 markers, with no expression of keratins AE1/AE3. This confirmed the diagnosis of localized melanoma. Notably, the resection margins were clear of neoplastic involvement.

The patient, discharged on postoperative day 9, commenced second-line therapy with ipilimumab.

However, despite this, his clinical condition deteriorated, and the patient passed away in July 2018.

## Discussion

Primary endobronchial melanoma (PEBM), an uncommon variant of melanoma primarily localized to the bronchial region, constitutes about 0.01% of all lung tumours, highlighting its rarity, especially when of bronchial origin [5]. PEBM presents with a spectrum of symptoms, including cough, haemoptysis, and dyspnoea, which often mimic more common respiratory conditions. However, the most challenging aspect lies in differentiating primary bronchial melanoma from metastatic melanoma, given the peculiar ability of skin melanomas to spontaneously regress without residual traces of metastasis [6, 7].

Melanomas without an identifiable primary account for roughly 15% of cases, complicating diagnosis [1]. To address this issue, a set of histological criteria has been proposed to establish the diagnosis of primary malignant melanoma in the bronchus [8–10]. These criteria include histopathological features, the presence of a solitary lung tumor, the absence of a history of cutaneous, mucous membrane, or ocular melanoma, and the absence of any other detectable tumour at the time of diagnosis [8–10]. Our patient met all these criteria, strongly indicating PEBM.

The pathogenesis of PEBM remains unclear. Theories suggested that PEBM may arise from melanocytes that migrated during foetal development along the primitive respiratory tract from the pharynx to the oesophagus [11]. Alternatively, it may originate from areas of squamous metaplasia, where epithelial cells transdifferentiate into melanocytes [12–14]. The presence of melanocytes in the bronchopulmonary system is indeed unusual. Similar phenomena have been observed in the basal larynx of patients with chronic laryngitis, suggesting that irritated bronchi undergoing squamous metaplasia may harbour similar changes [15]. The rarity of melanocytes within the bronchopulmonary system and the phenomenon of squamous metaplasia in the bronchi under chronic inflammatory conditions hint at possible pathogenetic mechanisms.

PEBM’s primary nature is suggested by polypoid, intraluminal growth, in contrast to the disseminated pattern typical of metastases [16]. Nevertheless, the oncogenesis of primary endobronchial melanoma remains an intricate puzzle to solve. The scarcity of reported cases, along with the intriguing phenomenon of spontaneous regression in cutaneous melanomas, has hindered our understanding.

Prognosis is variable. While some patients present with advanced disease at diagnosis, leading to poor outcomes, others may achieve long-term survival following successful tumour resection [17]. As of the time of this report, neoadjuvant and adjuvant therapies, as well as radiotherapy, were unestablished treatment options for cutaneous melanoma as for PEBM.

**Fig. 4:**
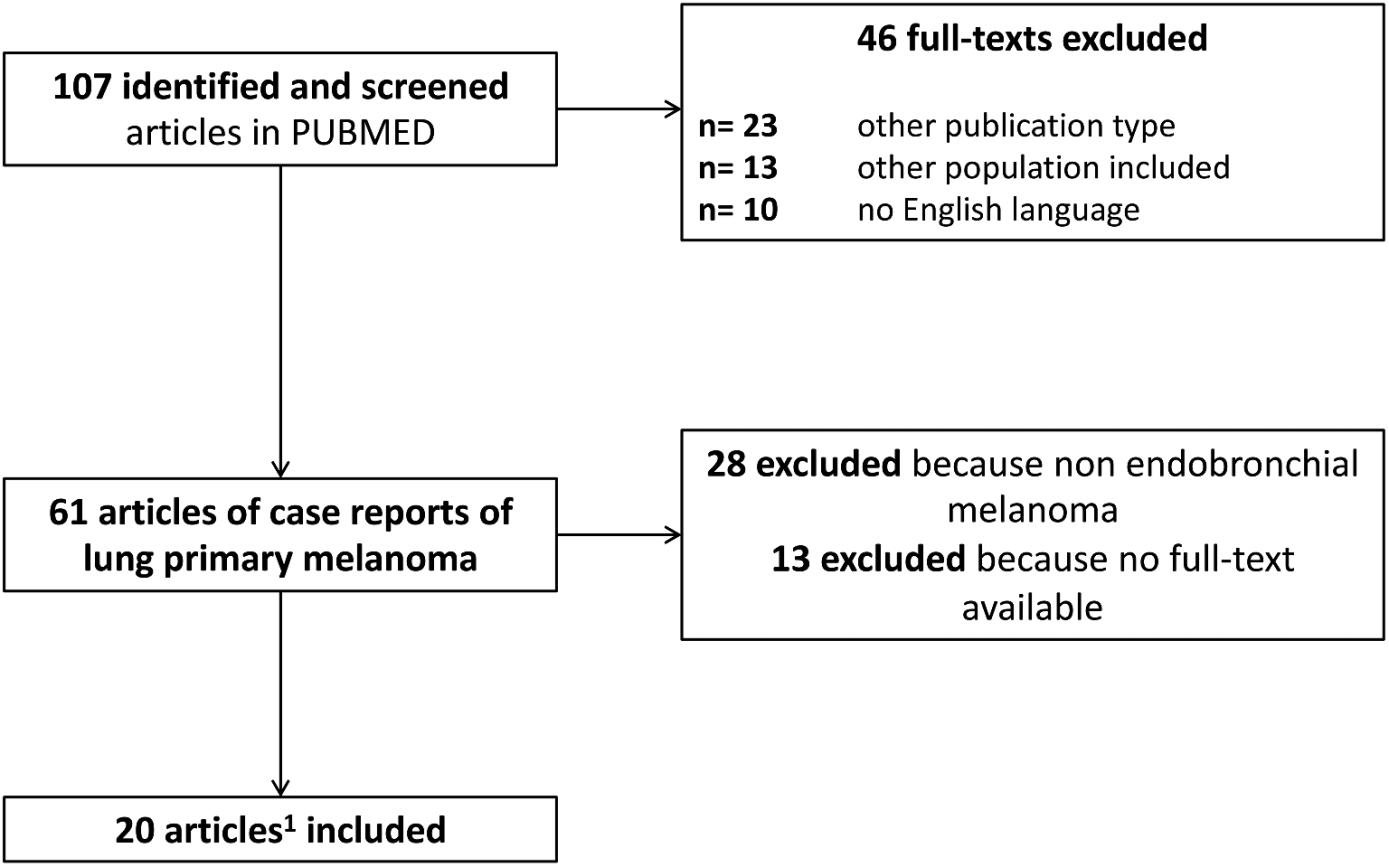
PRISMA flow-chart summarizing the process for the identification of the eligible articles. A total of 27 case reports were extracted from the included 20 articles

To elucidate this rarity, we conducted a systematic review of the literature, with the following research strategy: ((“melanoma“[Title]) AND (“primary“[Title])) AND ((((“endobronchial“[Title]) OR (“bronchus“[Title])) OR (“pulmonary“[Title])) OR (“lung“[Title])). We extracted data on 27 patients with PEBM, from a total of 20 articles (Fig. 4). The data, summarized in Table 1 [5, 11, 12, 16, 18–33], spanned from 1963 to 2023, revealing a wide range of outcomes. Notably, 12 patients succumbed to the disease, with a median survival of 14 months (range 2–32 months), 4 were alive with the disease with a median follow-up of 12 months (range, 4–30), and 9 were without evidence of disease (NED) with a median follow-up of 15 months (range 7-108). Histopathological diagnoses were primarily based on positive S100 and HMB45 staining, with one case exhibiting a BRAF mutation.

**Table 1:** Summary of the included case reports of endobronchial melanoma

Author	Year	Sex	Age	Involved Bronchus	Diagnosis	Histology	Treatment	Status	Survival time or follow-up (months)
Nigi A [18]	2021	M	71	LB	Transbronchial biopsy	HMB45, Melan-A, S100	Pembrolizumab	DOD	3
Bernal L [19]	2021	F	59	IB	Bronchoscopy	S100, HMB45	Pembrolizumab	NED	24
Wilson RW [5]	1997	M	71	LLL	Lobe	S100, HMB45	NA	NED	108
Wilson RW [5]	1997	M	45	LUL	Wedge	S100, HMB45	NA	AWD	4
Wilson RW [5]	1997	F	55	RUL	Lobe	S100, HMB45	NA	DOD	18
Wilson RW [5]	1997	M	52	LUL	Lobe	S100, HMB45	NA	DOD	32
Wilson RW [5]	1997	M	64	LUL	Lobe	S100, HMB45	NA	DOD	4
Wilson RW [5]	1997	M	48	LUL	Lobe	S100, HMB45	NA	DOD	14
Wilson RW [5]	1997	M	50	LUL	Lobe	S100, HMB45	NA	AWD	30
Hwang K-B [20]	2015	M	82	RLL	Bronchoscopy	S100, HMB45	No treatment	NA	NA
Yamamoto Y [21]	2017	F	61	S10	Wedge	S100, HMB45	DTIC, Anti-PD-1	DOD	15
Kyriakopoulos C [22]	2017	F	56	RUL	Bronchoscopy	MART-1, S100, HMB45	DTIC, IFNa2a, ipilimumab	DOD	5
Kim BC [23]	2023	M	62	Lingular bronchus	Bronchoscopy	S100, HMB45, BRAF V600E	lobe and lymphadenectomy, pembrolizumab, dabrafenib + trametinib	AWD	9
Mada PK [24]	2023	M	63	RUL	Bronchoscopy	SOX10	Nivolumab + Ipilimumab	DOD	2
Yabuki H [25]	2018	M	74	RB	Bronchoscopy	S100, HMB45	No treatment	NED	7
Taboada CF [12]	1972	M	40	LB	Bronchoscopy	Melanin pigment	Pneumonectomy	NED	36
Zhang X [26]	2015	M	60	LLB	Bronchoscopy	S100, HMB45, alfa-smooth muscle actin	Pneumonectomy and adjuvant chemotherapy	NED	18
Azuma Y [27]	2018	F	47	LB	Semi-rigid bronchoscopy	Pigmented mass	Nivolumab + Ipilimumab, Paclitaxel	DOD	3
dos Santos CL [28]	2013	F	62	LUL	Bronchoscopy	NA	RT, Dacarbazine	AWD	12
Adebonojo SA [29]	1979	F	55	RUL	Bronchoscopy	Melanin pigment	Adjuvant Melphalan	NA	NA
Bagwell SP [30]	1989	M	62	LUL	Bronchoscopy	NA	Lobe	DOD	2
Filosso PL [31]	2003	M	55	IB	Bronchoscopy	S100, HMB45	Pneumonectomy	NED	14
Farrell DJ [11]	1996	F	66	LLL	Bronchoscopy	NA	Lobe	NED	54
Dountsis A [32]	2003	F	41	RUL	Bronchoscopy	S100, HMB45	Pneumonectomy	NED	18
Barzò P [16]	1995	F	48	LUL	Bronchoscopy	NA	Pneumonectomy	NED	72
Barzò P [16]	1995	F	81	LB	Bronchoscopy	NA	Follow-up	DOD	5
Salm R [33]	1963	M	45	LLL	Autopsy	NA	Pneumonectomy	DOD	7

AWD: alive with disease; DOD: dead of disease; IB: intermediate bronchus; LB: left bronchus; LLB: lower left bronchus; LLL: left lower lobe; Lobe: lobectomy; NED: no evidence of disease; RB: right bronchus; RLL: right lower lobe; RT: radiotherapy; RUL: right upper lobe; S10: 10th segment

In conclusion, primary endobronchial melanoma remains a controversial entity, often necessitating an exclusion diagnosis. Like many malignant melanomas in other anatomical locations, radical surgical extirpation is considered mandatory, with or without regional lymph node dissection. Pneumonectomy with hilar lymph node dissection is often the treatment of choice, offering the best prospects for survival. However, with the advent of newer therapies, such as adjuvant (considering the PEBM a resectable metastasis of an unknown primary melanoma) or first-line (if the PEBM is unresectable) immunotherapy with nivolumab or the combination of nivolumab and ipilimumab [34–36], the treatment landscape for primary melanoma of the lung may evolve.

Given the small number of cases and the perplexing phenomenon of spontaneous regression in cutaneous melanomas, further research and data accumulation are needed to refine our understanding of this peculiar malignancy. With the evolution of therapeutic options, the management of primary endobronchial melanoma may become more effective, offering hope for improved outcomes.

## Data Availability

The data supporting the results reported in this manuscript are available upon request. Due to privacy considerations, individual patient data cannot be publicly shared. Requests for data access should be directed to the Corresponding Author and will be subject to any necessary ethical and privacy approvals.

## Abbreviations

CT: Body computed tomography
PEBM: primary endobronchial melanoma
PET: total body positron emission tomography
PMML: primary melanoma of the lung
